# Associations between Food Groups and Health-Related Quality of Life in Korean Adults

**DOI:** 10.3390/nu14173643

**Published:** 2022-09-03

**Authors:** Shamirah Nabbosa, Sunghee Lee

**Affiliations:** Department of Food and Nutrition, College of Health Science, Kangwon National University, Samcheok 25949, Korea

**Keywords:** EuroQol-5 Dimension (EQ-5D), health-related quality of life (HRQoL), food group, Korea National Health and Nutrition Examination

## Abstract

This cross-sectional study aimed to determine the associations between food groups and health-related quality of life (HRQoL). The data of 14,979 participants in the Korea National Health and Nutrition Examination Survey from 2016 and 2018 were examined. The HRQoL was assessed with EuroQol-5 Dimension. The 24-h recall test was used to examine the dietary intake of food groups. Males and females accounted for 13.79% and 21.62% of the low HRQoL groups. The males in the lowest tertile of the “vegetables” and “fish and shellfish” food groups were more likely to have a low HRQoL (odds ratio (OR), 95% confidence interval (CI) = 1.25 (1.05–1.65), 1.45 (1.12–1.89), respectively) than those in the highest tertile, whereas those in the lowest tertile of the “cereal and grain products” were less likely to have a low HRQoL (OR (95% CI) = 0.69 (0.52–0.91)). The females in the lowest tertile of the “vegetables” food group were more likely to have a low HRQoL (OR (95% CI) = 1.56 (1.17–3.01)) than those in the highest tertile. After adjusting for confounders indagated with not only dietary but also non-dietary factors such as stress, we found that low HRQoL was significantly associated with food groups of “vegetables”, “fish and shellfish”, and “cereal and grain products” among males and of “vegetables” among females.

## 1. Introduction

The World Health Organization described health-related quality of life (HRQoL) as a “comprehensive state of psychological, physiological, and social health, instead of just the absence of disease or infirmity” [1]. HRQoL is a multiple perspective that provides a broad concept of health in physical, mental, and social domains [2,3]. A study examining the magnitude of HRQoL in the Korean population demonstrated that 14.2% of males and 20.2% of females had difficulty in walking, 4.1% of males and 5.5% of females had difficulty in showering or dressing, 7% of males and 11.9% of females reported difficulties with regular activities, 18.1% of males and 27.7% of females reported pain and discomfort, and 9.0% of males and 15.8% of females reported feeling either worried or sad [4]. Moreover, a previous study has linked HRQoL to diabetes [3].

Regarding diet, HRQoL has been associated with diet-related factors such as diet quality [5], dietary patterns [6,7], malnutrition [8], Mediterranean diet [9,10], fruit and vegetable consumption [11], and food purchasing motivations [12]. Additionally, previous studies have demonstrated that poor diet quality characterized by high fat and sugar as well as insufficient intake of fruit and vegetables, was a contributing factor to reduced HRQoL [13,14,15,16]. Furthermore, depression, as one domain to determine psychological ill-health in HRQoL, has been associated with HRQoL [17]. Seafood such as fish and shellfish, dairy products, and fruit are excellent sources of tryptophan [9], an amino acid required for producing serotonin, what is known to be essential for having feelings of happiness [18]. 

However, to our best knowledge, no study on the relationship between food groups and HRQoL in a general population has yet been investigated. Moreover, it would be much easier to practice dietary guidelines based on food groups. Therefore, this cross-sectional study aimed to determine the associations between food groups and health-related quality of life (HRQoL) in a general population. 

## 2. Materials and Methods

### 2.1. Study Participants

**Figure 1:**
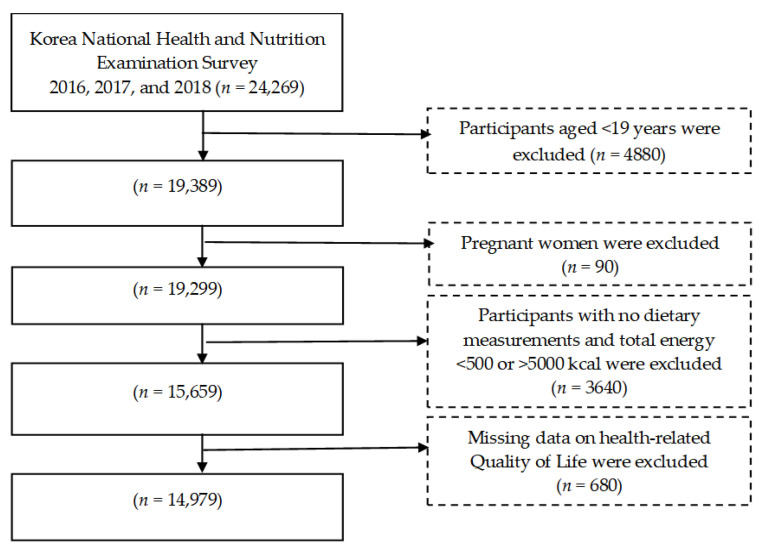
Flow chart of the study participants.

We used data from the Korea National Health and Nutrition Examination Survey (KNHANES) to investigate the associations between food groups and HRQoL among 14,979 Korean adults (6273 males and 8706 females) from a general population (Figure 1). We used data from the KNHANES 2016, 2017, and 2018 surveys. The KNHANES includes a representative population derived from a multi-step stratified cluster sampling procedure. All participants were informed about this study and signed a consent form. This study was approved by the Institutional Review Board of Korea Centers for Disease Control and Prevention (2018-01-03-P-A) and was waived due to the implementation of public well-being based on the Bioethics Act. For this study, the participants aged ≥19 years completed a 24-h recall assessment, a questionnaire of HRQoL, and a health behavior evaluation. 

### 2.2. Assessment of Health-Related Quality of Life (HRQoL)

HRQoL pertains to changes in physiological and psychological health status influenced by illness, aging, and nutritional status [19]. HRQoL can be measured using a variety of tools [20]. Among these, the EuroQol (EQ-5D) has been extensively used due to its cost-effectiveness; it also has been previously verified [21,22]. The EQ-5D has five domains including mobility, usual activities, self-care, anxiety/depression, and pain/discomfort, with three degrees of no, moderate, and severe difficulties [21]. These five components of the Korean EQ-5D were computed [20,22]. A higher EQ-5D score indicated a higher HRQoL [22,23]. 

### 2.3. Assessment of Dietary Intake

The intake of different food groups was assessed using a 24-h recall tool and the 18 food groups were classified including cereals and grains, potatoes and starches, sugars, seeds and nuts, mushrooms, fruits, vegetables, meat and their products, eggs, legumes and their products, fish and shellfish, seaweed, oils and fats, milk and dairy products, beverages, processed products, seasonings, and others.

### 2.4. Health Behavior Measurements

This study included the following health behavior measurements such as alcohol drinking (≥ four times per week, twice or thrice weekly, once monthly, or never), physical activity (≤ once per week, one or two days per week, three or four days per week, or ≥ five days per week), and stress recognition (very much, somewhat, a little, or rarely). The status of “being stressed” was determined if a participant answered with either “very much” or “somewhat”.

### 2.5. Statistical Analyses 

The weighted means and weighted frequencies were calculated. To compare the characteristics, we grouped the participants into two groups, either low or high HRQoL. Based on the distribution of EQ-5D scores, these two groups were EQ-5D ≤ 0.907 for the low HRQoL group as well as EQ-5D > 0.907 for the high HRQoL group. Furthermore, to investigate the associations between food groups and HRQoL, a logistic regression model was utilized to determine odds ratios (OR) and 95% confidence intervals (CIs) on the HRQoL status associated with the tertile groups of food group intakes. SPSS version 24.0 (IBM, Armonk, NY, USA) was used and the significance was determined as *p* < 0.05. 

## 3. Results

Table 1 presents 13.79% in males and 21.62% in females, showing a higher prevalence of low HRQoL among females. Both male and female participants of the low HRQoL were significantly older than those in the high HRQoL group (*p* < 0.001). Females in the low HRQoL had a higher body mass index (BMI) than those in the high HRQoL group (*p* < 0.001). Addtionally, in both males and females, the HRQoL was significantly associated with marital status (*p* < 0.001), education (*p* < 0.001), stress recognition (*p* < 0.001), drinking (*p* < 0.001), and exercise (*p* < 0.001). For both males and females, participants in the low HRQoL group showed higher prevalence rates of hypertension and diabetes than those in the high HRQoL group. For example, 38.93% of males with a low HRQoL were diagnosed with hypertension, compared with 17.84% of males with a high HRQoL. Of the females, 37.20% of the low HRQoL group were diagnosed with hypertension. Among males, the proportion of reporting frequent drinking (≥ 4 times a week) in the low HRQoL group was higher than that in the high HRQoL group (15.56% vs. 10.56%, *p* < 0.001).

Table 2 shows the intake levels of the different food groups among males according to the HRQoL groups. Among males, the “vegetables” and “fish and shellfish” intakes were significantly lower in the low HRQoL group than in the high HRQoL group (*p* = 0.048 and 0.013, respectively). In contrast, the intakes of “cereal and grain products”, “sugars and sweets”, and ”oils and fats” were significantly higher in the low HRQoL group than in the high HRQoL group (*p* = 0.039, 0.037 and 0.027, respectively). 

Table 3 presents the food group intakes among females according to the HRQoL groups among Korean adults. Females in the low HRQoL group consumed significantly fewer “vegetables”, “meats and their products”, “fish and shellfish”, and “processed products” than those in the high HRQoL group (*p*-value = 0.022, 0.045, 0.042, and 0.040, respectively). By contrast, females in the low HRQoL group reported a significantly higher intake of “sugars and sweets”, as compared to those in the high HRQoL group (*p*-value = 0.039).

Table 4 lists the consumption amounts of food groups and the weighted odds ratios of the low HRQoL group associated with the tertile ranges of food groups among the males, after adjusting for age, energy intake, body mass index, marital status, education, diabetes, hypertension, stress, drinking status, smoking, and exercise. As compared to males in the highest tertile of “vegetables” and “fish and shellfish” intake, males in the lowest tertile of “vegetables” and “fish and shellfish” intakes were 1.25 times and 1.45 times likely to have a low HRQoL (OR (95% CI) = 1.25 (1.05–1.65) and 1.45 (1.12–1.89), respectively). Additionally, males in the lowest tertile of “cereal and grain products” were 0.69 times less likely to have a low HRQoL (OR (95% CI) = 0.69 (0.52–0.91)).

Table 5 demonstrates the weighted odds ratios of the low HRQoL group associated with the tertile range of food group intakes among the females, after adjusting for those confounding factors. As compared to females in the highest tertile of the “vegetables” food group, females in the lowest tertile of the “vegetables” food group were 1.56 times more likely to have a low HRQoL (OR (95% CI) = 1.56 (1.17–3.01)). 

## 4. Discussion

In this cross-sectional study, we found significant associations of a low HRQoL with “vegetables”, “fish and shellfish”, and “cereal and grain products” food groups in males and only “vegetables” food group in females, after adjusting for age, energy, BMI, education, marital status, hypertension, diabetes, stress, drinking, smoking, and exercise. In particular, males in the lowest tertile of “fish and shellfish” and ”vegetable” food groups increased the likelihood of having a low HRQoL, and females in the lowest tertile of the ”vegetables” food group were likely to increase the odds of having a low HRQoL. Furthermore, males in the lowest tertile of “cereal and grain products” reduced the likelihood of having a low HRQoL, as compared to those in the highest tertile. To our best knowledge, this is the first study to examine the associations between food groups and HRQoL in a general population. 

Previous studies showed consistent results on HRQoL linked to diet quality [5], dietary patterns [6,7], and food purchasing motivations [12]. Furthermore, several cross-sectional studies on the associations between “vegetables” intake and HRQoL demonstrated significant relationships, indicating the beneficial effects of antioxidants and fibers in vegetables [9,10,11,24,25]. These studies also presented stronger associations with mental health than with physical well-being [9]. Furthermore, a longitudinal study of 459 survivors of colorectal cancer showed that participants with high intakes of fruit and vegetables demonstrated less fatigue and better performance of physical functioning, which are components of HRQoL [26]. Moreover, a randomized trial in 271 adults reported that participants who received two sessions of nutritional counselling to increase fruit and vegetable intake, manifested a high HRQoL, particularly through better physical health [27]. Additionally, a high fruits and vegetables intake was shown to have a strong link to psychological well-being, one of the domains in HRQoL [15]. 

Consistent with our current significant associations between “fish and shellfish” and HRQoL, a few studies have reported that “fish and shellfish” as a good protein source, was associated with improved HRQoL [5,8,9]. Interestingly, even after adjusting for omega-3 and -6 fatty acids, ”fish and shellfish” intake was reported to have a significantly positive association with physical well-being of HRQoL among a general population [28]. Sufficient protein intake from ”fish and shellfish” may provide enough essential amino acids to have a beneficial effect on musculoskeletal movements [29]. Indeed, higher adherence to the Mediterranean diet, consisting of predominately fruit and vegetables, olive oil, and fish and shellfish, was associated with a higher HRQoL score [16,24,25].

Underlying mechanisms on HRQoL including physical and mental well-being, as well as social aspects of functioning associated with food groups, have not been well elucidated. However, scientific evidence from previous studies have attempted to explain the associations via the beneficial effects of antioxidants, fiber, serotonin, and amino acids. For example, large consumptions of vitamins, minerals, and essential amino acids from vegetables, fish, and shellfish have been shown to help lead to increasing the production of serotonin of the brain [15]. Additionally, green leafy vegetables were determined to include high levels of antioxidants that protect the nervous system from free radical damage [14,15,30]. In this regard, a higher intake of vegetables and fruits have been indicated to be associated with higher HRQoL scores [14], reduced rates of depression [31], and lower levels of stress [32]. Besides, not only “vegetables” but also “fish and shellfish” were shown to be substantially related to a decreased association with depression [11,33]. Additionally, even though there is little evidence regarding “depression and anxiety” as one of five domains of HRQoL, previous research suggested that protein-derived amino acids, provided from “fish and shellfish”, may have been affected to mental health [34]. 

Our study has several strengths and limitations. Firstly, the key strength was to use the KNHANES data that have a representative sample of the general population, which allows us to have generalizability. Secondly, we were able to utilize the adjustment for potential confounding variables such as sex, age, energy intake, BMI, exercise, drinking and smoking habits, stress, education, marital status, hypertension, and diabetes. Despite these strengths, this study within a cross-sectional design had the limitation of having a difficulty in establishing a causality. Future longitudinal studies are suggested.

## 5. Conclusions

In conclusion, in this cross-sectional study, we found significant associations with the food groups “vegetables”, “fish and shellfish”, and “cereal and grain products” in males, and “vegetables” in females, with a low HRQoL, even after adjusting for possible confounding factors. Further studies are suggested to determine longitudinal associations between food groups and HRQoL.

## Figures and Tables

**Table 1:** Demographic characteristics according to the HRQoL stratified by sex among Korean adults (*n* = 14,979).

		Males			Females		
		(*n* = 6273)			(*n* = 8706)		
		HRQoL			HRQoL		
		Low	High		Low	High	
		(*n* = 1108)	(*n* = 5165)		(*n* = 2174)	(*n* = 6532)	
		13.79%	86.21%	*p*-Value	21.62%	78.38%	*p*-Value
Age (years)		56.38 ± 0.72	44.84 ± 0.29	<0.001	58.77 ± 0.54	45.46 ± 0.25	<0.001
Body Mass Index (kg/m^2^)		24.36 ± 0.14	24.58 ± 0.06	0.161	24.20 ± 0.09	23.05 ± 0.06	<0.001
Marital status	Married	77.48	70.59	<0.001	87.97	79.84	<0.001
	Single	22.52	29.4		12.02	20.16	
Education	Elementary	25.09	7.37	<0.001	45.58	11.89	<0.001
	Middle school	13.55	7.11		12.43	7.94	
	High school	33.83	36.67		23.18	36.26	
	University	27.53	48.84		18.80	43.9	
Hypertension	No	61.07	82.16	<0.001	62.79	86.64	<0.001
	Yes	38.93	17.84		37.20	13.36	
Diabetes	No	81.97	93.64	<0.001	84.73	95.14	<0.001
	Yes	18.02	6.36		15.27	4.86	
Stress recognition				<0.001			<0.001
	Very much	11.53	2.58		11.77	3.69	
	Somewhat	27.69	20.78		30.2 1	22.13	
	A little	46.48	60.33		45.12	59.49	
	Rarely	14.29	16.29		12.89	14.69	
Drinking Status				<0.001			<0.001
	<1/month	36.77	24.09		56.36	45.66	
	1–4/month	30.01	39.87		32.27	38.96	
	2–3/week	17.66	25.48		8.02	12.28	
	≥4/week	15.56	10.56		3.35	3.09	
Smoking Status				0.044			0.055
	Current	39.34	42.17		40.51	30.92	
	Previous	4.92	5.88		13.16	15.93	
	Never	55.74	51.96		46.33	53.16	
Exercise				<0.001			<0.001
	<1/week	76.33	65.16		87.12	80.79	
	1–2/week	7.25	11.75		4.43	7.83	
	3–4/week	7.27	11.91		4.77	6.62	
	≥5/week	9.16	11.19		3.68	4.75	

HRQoL, health-related quality of life.

**Table 2:** Food group intakes of males according to HRQoL among Korean adults (*n* = 6273).

	Low HRQoL (*n* = 1108)	High HRQoL (*n* = 5165)	*p*-Value
Cereal and grain products	303.38 ± 7.53	290.92 ± 6.22	0.039
Potatoes and starches	63.34 ± 7.95	69.24 ± 7.47	0.332
Sugars and sweets	11.49 ± 1.19	9.23 ± 0.89	0.037
Legumes and their products	58.82 ± 7.53	57.36 ± 5.62	0.808
Seeds and nuts	7.78 ± 1.99	0.37 ± 1.46	0.684
Vegetables	334.22 ± 11.84	350.83 ± 10.43	0.048
Mushrooms	9.41 ± 2.68	10.81 ± 2.21	0.511
Fruits	186.16 ± 18.22	193.21 ± 14.82	0.628
Meats and their products	190.79 ± 10.62	180.92 ± 9.00	0.253
Eggs	45.95 ± 3.92	47.82 ± 3.08	0.547
Fish and shellfish	120.49 ± 9.98	141.67 ± 9.77	0.013
Seaweed	50.46 ± 8.58	55.88 ± 7.82	0.396
Milk and dairy products	184.80 ± 20.46	178.14 ± 13.82	0.682
Oils and fats	8.92 ± 0.79	7.49 ± 0.44	0.027
Beverages	514.71 ± 30.44	529.91 ± 23.25	0.513
Seasonings	41.51 ± 1.96	40.66 ± 1.54	0.571
Processed products	150.75 ± 21.43	112.19 ± 16.02	0.056
Others	8.14 ± 5.33	10.25 ± 3.72	0.716

Means ± standard error (S.E.); adjusted for age, energy intake, body mass index, marital status, education, hypertension, diabetes, stress, drinking, smoking, and exercise.

**Table 3:** Food group intake of females according to HRQoL among Korean adults (*n* = 8706).

	Low HRQoL (*n* = 2174)	High HRQoL (*n* = 6532)	*p*-Value
Cereal and grain products	175.59 ± 12.22	163.92 ± 12.09	0.220
Potatoes and starches	73.28 ± 19.67	63.96 ± 14.86	0.512
Sugars and sweets	9.18 ± 1.46	6.77 ± 1.42	0.084
Legumes and their products	54.65 ± 13.76	44.42 ± 12.92	0.247
Seeds and nuts	20.55 ± 8.68	19.64 ± 8.54	0.749
Vegetables	229.89 ± 21.22	262.45 ± 18.58	0.022
Mushrooms	11.62 ± 5.33	11.27 ± 4.60	0.923
Fruits	161.73 ± 39.04	157.60 ± 36.59	0.881
Meats and their products	92.61 ± 15.75	117.39 ± 14.45	0.045
Eggs	52.83 ± 7.69	44.81 ± 6.96	0.125
Fish and shellfish	100.37 ± 21.15	118.60 ± 20.38	0.042
Seaweed	10.59 ± 13.30	4.17 ± 15.93	0.623
Milk and dairy products	221.93 ± 42.87	229.66 ± 41.57	0.767
Oils and fats	7.97 ± 1.17	6.61 ± 1.27	0.069
Beverages	770.78 ± 90.25	747.99 ± 82.89	0.700
Seasonings	30.32 ± 4.43	30.41 ± 4.06	0.973
Processed products	146.33 ± 39.29	191.72 ± 44.84	0.040
Others	2.43 ± 1.30	5.21 ± 2.10	0.165

Means± S.E.; adjusted for age, energy intake, body mass index, marital status, education, hypertension, diabetes, stress, drinking, smoking, and exercise.

**Table 4:** Weighted odds ratios of a low HRQoL associated with tertile ranges of food group intake among Korean males (*n* = 6273).

	Consumption Amount (g)		
	OR (95%CI)		
	T1	T2	T3
Cereal and grain products	0.63–195.55	195.56–303.47	303.48–1493.68
	0.69 (0.52–0.91)	0.80 (0.62–1.04)	1.00 (Ref)
Potatoes and starches	0.02–13.96	13.97–53.03	53.04–1591.60
	1.09 (0.75–1.57)	1.22 (0.84–1.75)	1.00 (Ref)
Sugars and sweets	0.00–2.10	2.20–7.20	7.30–260.00
	0.86 (0.65–1.15)	0.86 (0.65–1.13)	1.00 (Ref)
Legumes and their products	0.03–10.19	10.20–39.30	39.40–2128.99
	1.04 (0.77–1.39)	1.08 (0.82–1.42)	1.00 (Ref)
Seeds and nuts	0.00–0.50	0.60–2.50	2.60–1831.20
	0.97 (0.72–1.29)	1.01 (0.76–1.34)	1.00 (Ref)
Vegetables	0.28–194.39	194.40–350.60	350.70–2531.55
	1.25 (1.05–1.65)	1.19 (0.94–1.51)	1.00 (Ref)
Mushrooms	0.00–1.97	1.98–9.47	9.48–332.00
	1.18 (0.79–1.77)	1.09 (0.70–1.70)	1.00 (Ref)
Fruits	0.00–95.10	95.20–288.13	288.14–3204.17
	1.03 (0.76–1.38)	1.01 (0.75–1.37)	1.00 (Ref)
Meats and their products	0.24–50.76	50.77–129.87	129.88–1877.70
	0.92 (0.67–1.25)	1.14 (0.87−1.49)	1.00 (Ref)
Eggs	0.07−18.78	18.79–52.00	52.10–761.35
	1.04 (0.72–1.48)	0.89 (0.64–1.23)	1.00 (Ref)
Fish and shellfish	0.01–25.91	25.92–118.82	118.83–3042.71
	1.45 (1.12–1.89)	1.44 (1.11–1.86)	1.00 (Ref)
Seaweed	0.00–4.00	4.10–22.00	22.10–1510.17
	1.20 (0.86–1.68)	1.20 (0.87–1.65)	1.00 (Ref)
Milk and dairy products	0.07–102.40	102.50–208.00	208.10–1664.00
	0.94 (0.61–1.44)	0.87(0.56–1.34)	1.00 (Ref)
Oils and fats	0.00–2.20	2.30–6.70	6.80–230.00
	0.84 (0.63–1.11)	0.78 (0.58–1.05)	1.00 (Ref)
Beverages	0.00–35.00	35.10–355.20	355.30–7128.00
	1.24 (0.91–1.69)	1.25 (0.94–1.67)	1.00 (Ref)
Seasonings	0.00–15.95	15.96–36.37	36.38–874.69
	1.15 (0.88–1.50)	0.89 (0.69–1.13)	1.00 (Ref)
Processed products	0.05–40.98	40.99–142.90	143.00–1107.13
	0.56 (0.25–1.22)	0.38 (0.18–0.78)	1.00 (Ref)
Others	0.02–0.38	0.39–2.29	2.30–282.79
	1.25 (0.80–1.31)	1.29 (0.70–1.49)	1.00 (Ref)

Adjusted for age, energy intake, body mass index, marital status, education, hypertension, diabetes, stress, drinking, smoking, and exercise. OR, odds ratio; CI, confidence interval; Ref, reference; T1, first tertile; T2, second tertile; T3, third tertile.

**Table 5:** Weighted odds ratios of a low HRQoL associated with tertile ranges of food group intake among Korean females (*n* = 8706).

	Consumption Amount (g)		
	OR (95%CI)		
	T1	T2	T3
Cereal and grain products	0.32–195.56	195.57–303.47	303.48–1369.02
	0.59 (0.32–1.09)	0.61 (0.34–1.09)	1.00 (Ref)
Potatoes and starches	0.01–14.00	14.10–53.04	53.05−1888.50
	1.09 (0.52–2.34)	0.81 (0.39–1.67)	1.00 (Ref)
Sugars and sweets	0.00−2.10	2.20–7.20	7.30−292.10
	0.81 (0.46–1.44)	1.13 (0.70–1.81)	1.00 (Ref)
Legumes and their products	0.02−10.20	10.30–39.24	39.25−1319.95
	0.96 (0.52−1.76)	0.52 (0.26–1.04)	1.00 (Ref)
Seeds and nuts	0.00–0.50	0.60−2.50	2.60–603.59
	0.91 (0.52–1.59)	0.92 (0.49–1.71)	1.00 (Ref)
Vegetables	0.03−194.35	194.36−350.56	350.57–2663.71
	1.56 (1.17–3.01)	1.50 (0.81–2.96)	1.00 (Ref)
Mushrooms	0.00−1.97	1.98−9.47	9.48–340.65
	0.88 (0.40–1.90)	0.72 (0.35–1.50)	1.00 (Ref)
Fruits	0.00–95.10	95.20–288.36	288.37–4320.00
	0.86 (0.46–1.61)	0.91 (0.47–1.74)	1.00 (Ref)
Meats and their products	0.00–50.74	50.75–129.88	129.89–1321.53
	1.84 (0.96–3.55)	1.45 (0.77- 2.74)	1.00 (Ref)
Eggs	0.03 −18.77	18.78–52.00	52.10–442.63
	0.66 (0.35–1.26)	0.87 (0.44–1.70)	1.00 (Ref)
Fish and shellfish	0.00–25.94	25.95–118.84	118.85–1677.69
	1.01 (0.55–1.85)	1.12 (0.63–1.99)	1.00 (Ref)
Seaweed	0.00–4.00	4.10–22.05	22.06–1403.80
	1.69 (0.80–3.05)	0.84 (0.41–1.71)	1.00 (Ref)
Milk and dairy products	0.03–102.20	102.30–208.00	208.10–2080.00
	1.55 (0.79–3.05)	1.27 (0.58–2.77)	1.00 (Ref)
Oils and fats	0.00–2.20	2.30–6.70	6.80–139.20
	0.53 (0.29–1.08)	0.87 (0.55–1.39)	1.00 (Ref)
Beverages	0.00–35.00	35.10–355.20	355.30–4096.60
	1.25 (0.67–1.86)	1.49 (0.91–2.46)	1.00 (Ref)
Seasonings	0.02–15.97	15.98–36.39	36.40–409.12
	1.12 (0.67–1.86)	1.49 (0.91–2.46)	1.00 (Ref)
Processed products	0.03–40.98	40.99–142.90	143.00–853.59
	1.36 (0.80–1.68)	1.09 (0.37–1.72)	1.00 (Ref)
Others	0.01–040	0.50–2.15	2.16–540.00
	1.60 (0.20–1.12)	0.04 (0.01–1.73)	1.00 (Ref)

Adjusted for age, energy intake, body mass index, marital status, education, hypertension, diabetes, stress, drinking, smoking, and exercise.

## Data Availability

Not applicable.
